# Susceptibility of Human B-Lymphoblastoid Cells to Shiga Toxin Intoxication Homologues

**DOI:** 10.3390/microorganisms14071505

**Published:** 2026-07-10

**Authors:** Alfredo G. Torres, Alexander J. Badten, Susana Oaxaca-Torres, Itziar Chapartegui-González, Ennzo Ortega, Rama R. Atitkar, G. Jilani Chaudry, Carlton C. Brinkley, Angela Melton-Celsa

**Affiliations:** 1 Department of Biomedical Sciences, Meharry Medical College, Nashville, TN 37208, USA; 2 Department of Microbiology and Immunology, University of Texas Medical Branch, Galveston, TX 77555, USA; 3 Institute for Translational Sciences, University of Texas Medical Branch, Galveston, TX 77555, USA; 4 Division of Clinical Infectious Diseases, Department of Medicine Huddinge, Karolinska Institutet, 14152 Stockholm, Sweden; 5 Department of Microbiology and Immunology, Uniformed Services University of the Health Sciences, Bethesda, MD 20814, USA; 6 Henry M. Jackson Foundation for the Advancement of Military Medicine, Inc., Bethesda, MD 20817, USA; 7 59 MDW Chief Scientist’s Office, Lackland Air Force Base, San Antonio, TX 78234, USA

**Keywords:** Shiga toxin, Gb3, globotriaosylceramide, B-lymphoblastoid cells

## Abstract

Shiga toxins (Stx), produced by Stx-producing *Escherichia coli* (STEC), are known to target Gb3-expressing cells, contributing to organ pathology such as in the kidney and brain. However, the sensitivity of human B-lymphoblastoid cell lines to Stx2 and their Gb3 expression profiles remain poorly understood. In this preliminary study, we assessed the susceptibility of human B-lymphoblastoid cell lines to Stx2 and identified distinct resistance and sensitivity patterns. Eight representative lines were further analyzed for Gb3 expression by mass spectrometry and flow cytometry. Susceptible cell lines (e.g., GM02473 and GM07019) displayed significantly higher total and membrane-associated Gb3 levels, while resistant lines had lower or undetectable Gb3. Exosomal Gb3 quantification revealed similar expression trends, contradicting the hypothesis that Gb3-positive exosomes neutralize Stx2. Interestingly, resistant cell line GM17658 showed discordant total and exosomal Gb3 levels. Immunofluorescence microscopy and flow cytometry revealed heterogeneous Gb3 expression within cell lines, with susceptible lines having a higher proportion of Gb3-positive cells. These findings suggest that Stx2 susceptibility is associated with Gb3 expression frequency rather than intensity and raise the possibility that Gb3-positive exosomes might contribute to toxicity. Future studies need to validate the role of exosomal Stx2 transfer and the functional impact of variable levels of Gb3-positive versus Gb3-negative subpopulations in toxin response.

## 1. Introduction

Globotriaosylceramide (Gb3, also known as CD77) plays a critical role in the binding and internalization of Shiga toxin (Stx) into host cells [1,2]. This receptor is a glycosphingolipid expressed on the surface of various mammalian cell types, including endothelial cells in the kidneys and intestines [3]. The B subunit of Stx specifically binds to Gb3, facilitating the toxin’s attachment to cell membranes. This interaction enables the internalization of the toxin, which ultimately leads to inhibition of protein synthesis and activation of cellular damage pathways [4]. Stx type 1 and/or 2 are produced by certain strains of *Escherichia coli* that are lysogenized with *Stx*-converting bacteriophages. Such Stx-producing *E. coli* (STEC), including O157:H7 outbreak strains, are responsible for severe gastrointestinal diseases and systemic sequela, including hemolytic uremic syndrome (HUS) [5,6]. Understanding the role of the Gb3 receptor in Stx binding has been essential for developing targeted therapeutic strategies to prevent or mitigate toxin-related pathologies [7].

Importantly, cells that lack or express only minimal levels of Gb3 on their surface are generally resistant to the cytotoxic effects of Stx, because this receptor serves as the primary binding site for the toxin [8,9]. Therefore, cells with minimal or absent Gb3 expression exhibit natural resistance to the damage mediated by Stx [8,10]. Once Stx binds Gb3, it is internalized and retrogradely transported to the endoplasmic reticulum and ribosomes, where the enzymatic A subunit of Stx exerts its cytotoxicity by catalytically inactivating the 23S rRNA of the large ribosomal subunit. This toxin action inhibits protein synthesis and induces cell death [11]. Several types of host cells fall into this category of having limited or no Gb3 and demonstrate natural resistance [12], including (a) most mammalian epithelial cells, such as those lining the respiratory tract; (b) neuronal cells; (c) fibroblasts; (d) erythrocytes; and (e) platelets.

The resistance of these cell types underscores the essential role of Gb3 as the mediator of Stx’s toxic effects. In contrast, cells that do express Gb3, such as certain renal and vascular endothelial cells, are susceptible to Stx-induced damage, which can result in severe complications like HUS [5,6]. Previous studies showed that B lymphocytes isolated from human samples and several B lymphoblast lines, but not bone marrow or pre-B cells, were sensitive to Stx and expressed Gb3 [13,14]. Further, Stx susceptibility/resistance and Gb3 expression have been reported in some Burkitt’s lymphoma cells and germinal centers [15]. The finding that certain B cell subsets express Gb3 led to some research exploring the role of Gb3 in B cell maturation [16,17,18] and the potential use of Stx or moieties containing the Stx binding B subunit pentamer as possible treatments for Gb3-positive cancers [19,20,21]. In this context, our study focused on analyzing the relative sensitivity to Stxs of human B-lymphoblastoid cells isolated from a variety of sources including those with no known pathology, because it is important to determine whether expression levels of Gb3 correlate with sensitivity or resistance to Stx. Furthermore, since human lymphoblastoid cells have emerged as a promising model system in the study of drug response, it is important to understand the biological differences among the various sources of B cells [22].

## 2. Materials and Methods

### 2.1. Ethical Statement

The study received IRB approval from 59 MDW with the protocol number FWH20220111N, and it was considered non-human research as defined by DoD regulation 32 CFR 219 and FDA regulation 21 CFR 56. At UTMB, the protocol was reviewed by the Institutional Biosafety Committee and received approval under NOU# 2018035. The cell lines used in this study were obtained from the NHGRI Sample Repository for Human Genetic Research at the Coriell Institute for Medical Research.

### 2.2. B Cell Line Culture and Stxs

B-lymphoblastoid cells were purchased from the Coriell Institute (https://www.coriell.org/ (Accessed on 20 October 2024)) and tested as described. The cell lines used are listed in Table 1. The cells were maintained at 37 °C with 5% CO_2_ in complete RPMI (Gibco, Waltham, MA, USA), supplemented with 1 mM sodium pyruvate, 1× non-essential amino acids, penicillin (100 U/mL), streptomycin (100 µg/mL) and 10% heat-inactivated FBS. Stx1, Stx2, and a subtype of Stx2, Stx2d, were purified as described previously [7,23].

For toxicity assays, cells were seeded at approximately 6 × 10^5^ cells/mL and distributed as 3 × 10^4^ cells/well in a total volume of 50 μL. Each cell line was seeded on a 96-well plate in triplicate for each condition and incubated overnight at 37 °C at 5% CO_2_. The day after the seeding, cells were exposed to 10-fold dilutions of purified toxin stocks. Untreated cells were used as controls. The plates were then incubated for 72 h at 37 °C with 5% CO_2_.

After the incubation time, CellTiter-Glo^®^ Luminescent Cell Viability Assay (Promega, Madison, WI, USA) was used to measure the level of ATP as an indicator of cell viability following the manufacturer’s protocol. Luminescence was measured by a luminometer (GloMax, Promega or Synergy H1, BioTek, Winooski, VT, USA). The 50% cytotoxic dose (CD_50_) was estimated as the concentration of toxin required to kill 50% of the cells.

For data analysis and normalization, the reads from the toxin untreated controls were considered to reflect 100% viability, and the corresponding values from each cell line that received the toxin dilutions were normalized to the untreated controls.

For the binding and imaging assays, B cells were pelleted by centrifugation and resuspended in phosphate-buffered saline (PBS). Then, 0.5 mL of the suspension at 10^5^–10^6^ cells/mL was added to a glass coverslip in a 24-well plate and incubated at room temperature for 30 min. The PBS was then removed, and the cells were fixed with 0.5 mL formalin for 10 min. The formalin was removed, and the cells were washed with PBS. Next, the cells were overlaid with 0.5 mL Stx1 and Stx2 (1 μg/mL; the prototypes Stx1a and Stx2a were used for all the experiments) and incubated at room temperature for about 1 h. The toxin mixture was removed, the coverslip was washed and overlaid with blocking solution (10% normal rabbit serum, Thermo Fisher, Waltham, MA, USA) for 30 min; then, it was washed again and overlaid with both rabbit polyclonal anti-Stx1 and monoclonal anti-Stx2 (11E10) diluted 1:2000 in 10% normal rabbit serum for 1 h. The coverslip was washed again and incubated overnight at 4 °C with secondary antibodies (Alexa Fluor 633 goat anti-rabbit IgG and Alexa Fluor 488 donkey anti-mouse IgG, both from Thermo Fisher) diluted 1:2000 in 10% normal rabbit serum. Next, the coverslips were washed three times with PBS and mounted onto glass slides using Slowfade 4′,6-diamidino-2-phenylindole (DAPI, Thermo Scientific, Carlsbad, CA, USA).

### 2.3. Mass Spectrometry Absolute Quantification of Total Gb3 Receptor Expression and Exosome-Expressed Gb3 Receptor

We cultured eight representative human B-lymphoblastoid lines (GM00333, GM00607, GM02473, GM06989, GM07019, GM17197, GM17645, and GM17658) to ≥10^6^ cells/mL. The 10^6^ cells were collected into 1.5 mL tubes, which were centrifuged at 400× *g* for 10 min, and the supernatant was carefully pipetted off and discarded. The resulting pellet was then washed twice with PBS and stored at −80 °C until ready for mass spectrometry sample processing. We separately cultured the same cell lines in media containing exosome-depleted FBS (Thermo Fisher Scientific, Waltham, MA, USA) until cells reached ≥10^6^ cells/mL. Cells were then collected and centrifuged at 400× *g* for 10 min at 4 °C. One mL of culture supernatant was collected, and exosomes were isolated using the Total Exosome Isolation reagent (Thermo Fisher Scientific) according to the manufacturer’s directions. At the end of the protocol, exosome-containing pellets were reconstituted in 25 μL of PBS. The total protein concentration of the exosomes was measured using a MicroBCA kit (Thermo Fisher Scientific) according to the manufacturer’s instructions, and protein concentrations were used as a surrogate measure of exosome concentration. Exosomes were stored at −20 °C until ready for mass spectrometry sample processing.

Prior to the extraction of cells or exosomes, 10 μL of a mixture of isotopically labeled lipid standards containing 1:10 diluted UltimateSPLASH ONE (Avanti Polar Lipids, Alabaster, AL, USA) and 1:10 diluted SphingoSPLASH I (Avanti Polar Lipids) was added to the samples. The samples were then extracted using a modified methyl tert-butyl ether extraction (MTBE, LC/MS Grade, Fisher Chemical, Waltham, MA, USA) [24]. The organic phase was aspirated off and dried under nitrogen gas. The dried extracts were resuspended in 200 μL of dichloromethane/methanol (1:1, *v*/*v*) (DCM, HPLC grade, Thermo Scientific; MeOH, Optima LC/MS grade, Fisher Chemical).

To quantitate the Gb3 content, a standard curve was prepared with Gb3 18:1;O2/17:0 (Cayman Chemical, Ann Arbor, MI, USA) with dilutions from 1 to 5000 ng/mL. Each dilution was prepared in DCM/MeOH (1:1, *v*/*v*) with 10 μL of the same 1:10 diluted internal standards as the samples. Peak areas of the calibrants or samples were normalized to the peak area of the LPE 19:0[D5] internal standard as part of the UltimateSPLASH ONE mix to produce peak area ratios. Peak area ratios of the calibrants were plotted as a linear curve against the prepared Gb3 18:1;O2/17:0 concentrations. A linear regression produced the equation of a line used to calculate the concentrations of Gb3 within samples. Gb3 concentrations within exosomes were further normalized to the corresponding protein concentration as determined by the MicroBCA assay described above. For quantitative comparisons, all sample values were normalized to 10^6^ cells.

LC-MS/MS was performed with an Acquity Premier HPLC System (Waters, Milford, MA, USA) coupled to a QTRAP 6500 mass spectrometer (SCIEX, Toronto, ON, Canada). The Gb3 lipids were separated by HILIC chromatography on a Luna NH2 column (Phenomenex, Torrance, CA, USA, 3 μm, 150 × 4.6 mm). Mobile phases and LC conditions are the same as previously published for polar lipidomics analysis [25]. The mobile phases included (A) acetonitrile/water/hexane (92:6:2, *v*/*v*) + 10 mM ammonium acetate, pH 9.3, and (B) acetonitrile/water (1:1, *v*/*v*) + 10 mM ammonium acetate, pH 9.3. Gb3 lipids were detected using multiple reaction monitoring (MRM). The source conditions were optimized using the Analyst software (SCIEX, v1.7.3) Compound Optimization functionality. Q1 masses were detected as protonated adducts ([M + H]^+^), while the Q3 masses were detected as the long chain base fragment ion. MRM transitions were calculated for Gb3 lipids with either a ceramide or dihydroceramide base and a variety of fatty acid chain lengths from 16 to 24 (see Supplementary Table S1 for the full list of MRM transitions monitored). The source parameters included the following: declustering potential (DP) of 116 V, entrance potential (EP) of 10 V, collision energy (CE) of 65 V, collisional exit potential (CXP) of 18 V, curtain gas (CUR) of 30 psi, collisional activation dissociation (CAD) set to medium, ion spray voltage (IS) set to 5500 V, ion spray temperature (TEM) set to 425 °C, nebulizing gas (GS1) set to 50 psi, and heating gas (GS2) set to 55 psi. LCMS peak areas were integrated using MultiQuant software (v3.0.3, SCIEX). The total Gb3 amount and Gb3 species were plotted using R/RStudio (R v4.3.3, RStudio v2024.04.0) and the following R packages: data.table (v1.15.4) and ggplot2 (v3.5.1). Figures were created and correlation coefficients calculated with GraphPad Prism 11.

### 2.4. Flow Cytometry Assessment of Cell Membrane-Expressed Gb3

Eight representative human B-lymphoblastoid lines (GM00333, GM00607, GM02473, GM06989, GM07019, GM17197, GM17645, and GM17658) were grown to ≥10^6^ cells/mL. We then collected 10^6^ cells from each into separate 1.5 mL tubes. These were centrifuged at 400× *g* for 5 min at 4 °C into a pellet, and the pellet was washed twice with 100 µL PBS. Cells were then stained for 30 min at 4 °C with 100 µL of 1.25 µg/mL mouse anti-Gb3 IgG2b (TCI America, Portland, OR, USA). The cells were then washed again two times with PBS. Next, the cells were stained for 30 min at 4 °C with 100 µL of 1.25 µg/mL rat anti-mouse IgG2b-PE (BioLegend, San Diego, CA, USA) and 1:1000 diluted Zombie NIR (BioLegend) in the dark. The cells were washed again twice in PBS and subsequently fixed in Fixation Buffer (BioLegend) for 20 min in the dark. The cells were washed in PBS two more times before the pellets were finally reconstituted in 1 mL of FACS buffer (BioLegend). Samples were run on an LSR Fortessa flow cytometer (Becton, Dickinson and Company, Franklin Lakes, NJ, USA), and 20,000 cells were analyzed per sample. FlowJo v10.10 was used for all data analysis. Figure was created and correlation coefficient calculated with GraphPad Prism 11.

## 3. Results and Discussion

### 3.1. Stx2 Cytotoxicity Experiments with Human B Lymphoblastic Cell Lines

Stxs produced by STEC are released in the intestine of infected patients and transferred across the intestinal epithelium into the circulation. While Stx-mediated damage to Gb3-expressing cells, such as that found in the kidney and brain, is well documented [26], the sensitivity of B-lymphoblastic cells to Stx and the levels of the Gb3 receptor on those cells has not been studied to any appreciable degree.

**Figure 1:**
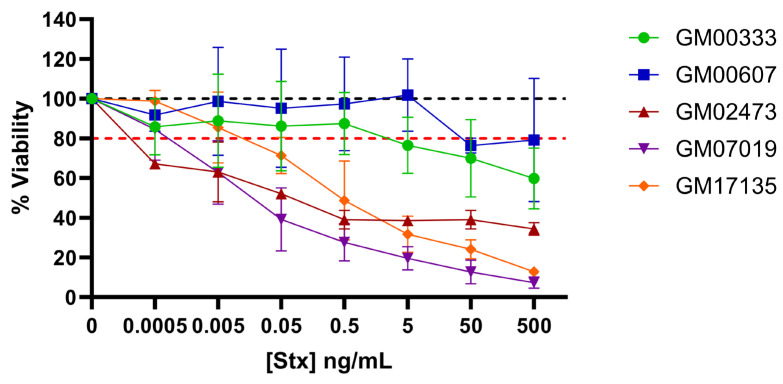
Stx2 cytotoxicity assay for selected B-lymphoblastoid cell lines. The cell lines GM00333, GM02473, GM17135, GM00607 and GM07019 were intoxicated (n = 3) with 10-fold serial dilutions of Stx2, and after 72 h, the viability was evaluated using CellTiter Glo. Viability of cells that received no Stx2 was set at 100%, and it was used as the normalization standard.

We initially obtained a set of five B-lymphoblastoid cells lines (GM00333, GM00607, GM02473, GM07019 and GM17135), which were used for an intoxication assay with Stx2. Three cell lines GM02473, GM07019, and GM17135 displayed various levels of sensitivity to the toxin, while B-lymphoblastoid cells GM00607 and GM00333 were resistant to Stx2 up to 1000 ng/mL and 1 ng/mL, respectively (Figure 1 and Table 1 for summary data).

**Figure 2:**
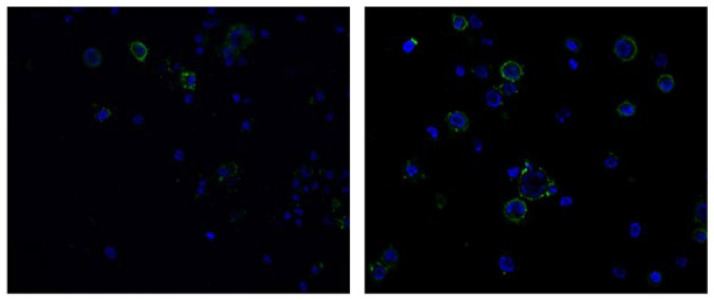
Stx2 binding to B-lymphoblastoid cell lines shown by immunofluorescence. GM04258 (**left panel**) and GM17154 (**right panel**) were overlaid with Stx2 (green). Representative images are shown. n ≥ 4.

To visualize Stx2 binding to the cells, we overlaid GM17154 (CD_50_ = 0.1 ng/mL) and GM04258 (CD_50_ = 2 ng/mL) with Stx2 and observed toxin binding by immunofluorescence (Figure 2). As expected, the susceptible cell line GM17154 exhibited markedly higher Stx binding than the more resistant cell line GM04258. We found that the binding pattern matched the cytotoxicity data in that GM17154 bound more Stx2 than GM04258. We also noted that, in both cell lines, only a subset of cells could bind Stx to a detectable level, and many cells completely lacked Stx binding. However, we are cognizant that while primary germinal center B cells readily express Gb3, some B-lymphoblastoid cell lines could downregulate the receptor due to the activating effects of Epstein–Barr virus during immortalization [27]. Therefore, additional cell lines were tested for susceptibility to Stx1 and Stx2d (Supplemental Figure S1), and we again noted a wide difference in susceptibility to the toxins. On some cell lines, there was considerable variation in susceptibility, for reasons that were not initially understood. However, we hypothesized that the differences we noted were due to differences in Gb3 levels and/or populations and how the toxins bound to the cells.

To understand why some human B-lymphoblastoid lines were susceptible to Stx2 while others were resistant, we first decided to explore whether the Stx2 receptor Gb3 is differentially expressed by two different methods (i.e., mass spectrometry and flow cytometry), to evaluate which provides the relative level of Gb3 expression in the susceptible versus the resistant cells in a quantitative way.

### 3.2. Mass Spectrometry Absolute Quantification of Total Gb3 Receptor Expression

We hypothesized that susceptible cells would have a higher expression of Gb3 on their surface than resistant cells, leading to an increase in Stx2 internalization. Therefore, we explored whether the Stx2 susceptibility of the immortalized human B-lymphoblastoid lines was correlated to the abundance of the Stx2 receptor Gb3. We cultured eight representative cell lines (GM00333, GM00607, GM02473, GM06989, GM07019, GM17197, GM17645, and GM17658) and performed lipid extraction on cell pellets, as described in the methods section.

**Figure 3:**
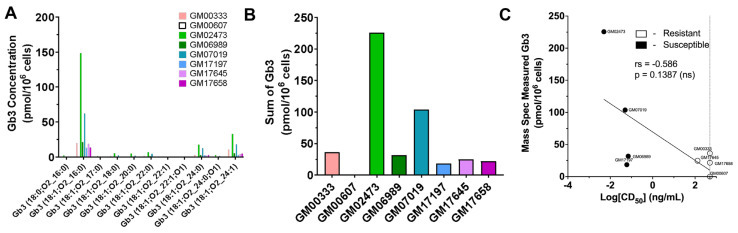
Mass spectrometry of human B-lymphoblastoid cell lines. Absolute quantification of individual Gb3 species (**A**) or total Gb3 (**B**) extracted from immortalized human B-lymphoblastoid lines was performed by LC-MS/MS. Individual lipid species are labelled according to their LIPID MAPS Structure Database shorthand designation (e.g., 18:1;O2 denotes a dihydroxy long-chain base) followed by the shorthand designation of the fatty acid chain. Spearman correlation coefficient and linear regression curve comparing LC-MS/MS measured Gb3 concentrations to the log (CD_50_) values from Table 1 (**C**).

Eleven distinct species of Gb3 were identified, though Gb3 (18:1;O2_16:0) was the dominant species for all cell lines that had detectable Gb3, ranging from 54 to 76% of the total Gb3 detected (Figure 3A). Most of the remaining Gb3 consisted of Gb3 (18:1;O2_24:0), ranging from 8 to 15% of the total, and Gb3 (18:1;O2_24:1), which ranged from 15 to 29%, though trace amounts of Gb3 (18:0;O2_16:0), Gb3 (18:1;O2_17:0), Gb3 (18:1;O2_18:0), Gb3 (18:1;O2_20:0), Gb3 (18:1;O2_22:0), Gb3 (18:1;O2_22:1), Gb3 (18:1;O2_22:1;O1), and Gb3 (18:1;O2_24:0;O1) were also detected (Figure 3A). GM02473 and GM07019, which had the highest measured levels of Gb3, had correspondingly high Stx2 susceptibility (Figure 1 and Figure 3B, Table 1). The Stx2 resistance of GM17645, GM17658, GM00333, and GM00607 are also consistent with their low levels of Gb3 (Table 1, Figure 3B). The Gb3 concentration exhibited a moderate correlation with the sensitivity to Stx2 (rs = −0.586, *p* = 0.1387), largely agreeing with our initial hypothesis. However, GM06989 and GM17197 were more susceptible to Stx2 than their Gb3 levels would suggest, hinting at another potential mechanism of susceptibility in these cell lines (Figure 1 and Figure 3B,C, Table 1). It is not currently understood why each cell line displayed different levels of Gb3, though it should be noted that prior studies have found that only germinal center B cells and some B cell lymphoma cells express Gb3 [18]. However, it is also possible that the variability may also result from immortalization of the B. cells [28]. Therefore, it may be worth exploring whether the expression of Gb3 in these cell lines is related to the phenotype of the B cells that were collected for immortalization or genetic/environmental factors related to the donor (see Table 1).

### 3.3. Mass Spectrometry Absolute Quantification of Exosome-Expressed Gb3 Receptor

We then explored whether exosomes displaying Gb3 might be playing a role in promoting Stx2 resistance by serving as a sort of “sponge” for Stx2. A similar phenomenon has recently been demonstrated using synthetic Gb3-coated exosomes as a potential therapeutic to neutralize the toxin [29], and human-derived exosomes from various sources are known to be able to harbor Gb3 [30,31]. We cultured the same eight representative cell lines as before, this time using exosome-depleted FBS to reduce assay interference from bovine-derived exosomes. Supernatants were collected after the cells had sufficiently grown, and exosomes were isolated and measured for total protein content. Lipids were then extracted from the exosome preparations and analyzed by LC-MS/MS.

**Figure 4:**
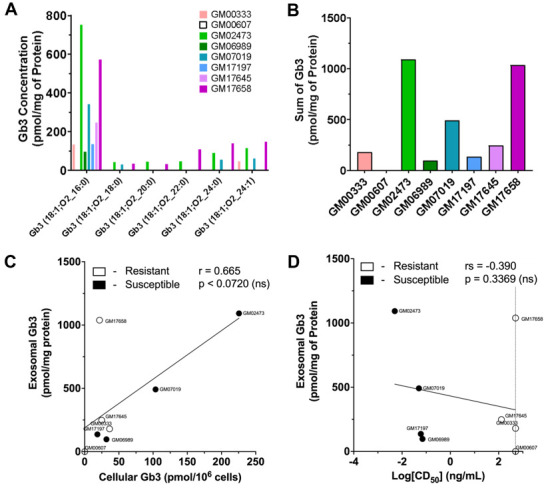
Mass spectrometry of exosomes. Absolute quantification of individual Gb3 species (**A**) or total Gb3 (**B**) extracted from exosomes derived from different immortalized human B-lymphoblastoid lines was performed by mass spectroscopy. Individual lipid species are labelled according to their LIPID MAPS Structure Database shorthand designation (e.g., 18:1;O2 denotes a dihydroxy long-chain base) followed by the shorthand designation of the fatty acid chain. Pearson correlation coefficient and linear regression curve comparing exosomal Gb3 content to the cellular Gb3 concentrations from Figure 3B (**C**). Spearman correlation coefficient and linear regression curve comparing exosomal Gb3 content to log (CD_50_) values from Table 1 (**D**).

In this experiment, only six species of Gb3 were identified, though the dominant species was again Gb3 (18:1;O2_16:0), ranging from 55 to 100% (Figure 4A). Contradicting our hypothesis, the exosome-expressed Gb3 concentrations shared a relatively similar profile with total cell Gb3 expression (r = 0.665, *p* < 0.0720), indicating that susceptible cells have elevated levels of both total Gb3 and exosome-expressed Gb3 (Figure 3B and Figure 4B,C). GM17658, a resistant cell line, was an exception to this, exhibiting relatively low cellular Gb3 but particularly high exosomal Gb3 concentrations (Figure 3B and Figure 4B). Importantly, there was only a weak statistically non-significant negative correlation between exosomal Gb3 and Stx2 CD_50_ values (rs = −0.390, *p* = 0.3369) (Figure 4D). Therefore, the evidence does not support a protective role for Gb3-coated exosomes in neutralizing the toxin, at least in these cell lines. Instead, it is possible that Gb3-expressing exosomes may bind to Stx2 and become internalized with the exosome, a mechanism worthy of future exploration. This phenomenon has previously been reported in a study that showed that Stx2-treated macrophages secrete Stx2-containing exosomes that are taken up by other cell types, which the authors speculated may contribute to HUS complications [32].

### 3.4. Flow Cytometry Assessment of Cell Membrane-Expressed Gb3

**Figure 5:**
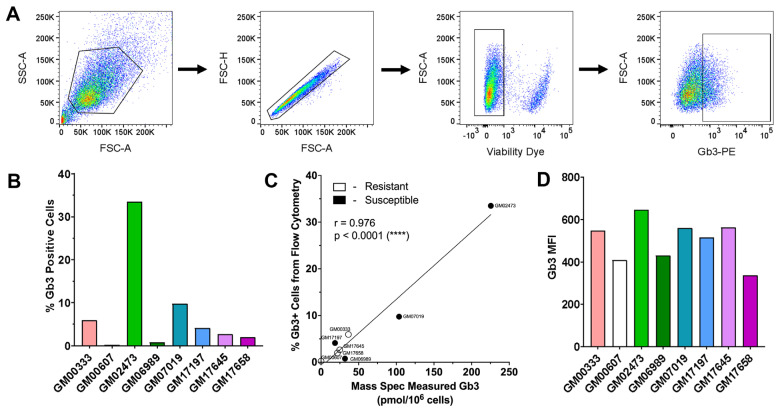
Gb3expression in representative human B-lymphoblastoid cells was performed by flow cytometry. Gating strategy (**A**). Percentage of total live cells that are Gb3+ (**B**). Pearson correlation coefficient and linear regression curve comparing the percentage of Gb3+ cells as determined by flow cytometry to the concentration of cellular Gb3 measured by LC-MS/MS from Figure 3B (**C**). Median fluorescence intensity (MFI) of the Gb3 signal in Gb3+ cells (**D**).

To confirm the mass spectrometry results and measure only the cell surface-expressed Gb3, we decided to employ a flow cytometric approach (Figure 5A). The same eight representative cell lines were incubated with mouse anti-Gb3 IgG2b and then an anti-IgG2b-PE secondary antibody to fluorescently stain the surface Gb3.

As suggested by the Stx binding assay (Figure 2), the Gb3 expression was not uniform within each cell line. Instead, there were distinct Gb3-negative and Gb3-postive populations (Figure 5A). Furthermore, the proportion of Gb3-positive cells differed considerably between the cell lines, and this profile correlated with the mass spectrometry results very strongly (r = 0.976, *p* < 0.0001) (Figure 3B and Figure 5B,C). Meanwhile, the median fluorescent intensity (MFI) of the Gb3-positive population was consistent between all cell lines, indicating there were not major differences in the relative abundance of membrane Gb3 between cell lines (Figure 5D). This suggests that the susceptible cell lines GM02473 and GM07019 may have a higher proportion of Stx2-sensitive cells, leading to a higher number of dead cells upon Stx2 treatment. At this time, we cannot conclude with certainty whether the Gb3-negative population is resistant to Stx2 treatment due to the presence of Gb3-positive cells in all lines assessed, except GM00607 (Figure 3B and Figure 5B). Future studies may utilize cell sorting to separate the Gb3-positive and negative cells independently and then treat them with the toxin. Furthermore, it should be investigated whether the presence of Gb3-positive cells increases the susceptibility of Gb3-negative cells to Stx2 via Stx2-loaded exosomes. Lastly, it remains poorly understood why individual human lymphoblastoid cell lines have such heterogenous expression of Gb3.

## 4. Conclusions

This preliminary study demonstrates that human B-lymphoblastoid cell lines exhibit a wide range of sensitivities to Stx2. Immunofluorescence, mass spectrometry, and flow cytometry confirmed generally that susceptibility correlates with the proportion of Gb3-positive cells, rather than the amount of Gb3 per cell. Interestingly, Gb3-expressing exosomes do not appear to neutralize Stx2 but may instead facilitate toxicity by transferring the toxin to otherwise resistant cells. Although the analysis was limited due to the availability of cells, these findings highlight the complexity of Stx2 interactions and suggest that both Gb3 expression and exosomes contribute to cellular susceptibility.

## Figures and Tables

**Table 1:** B-lymphoblastoid cell line differential susceptibility to Stx2.

Cell Line ^a^	Description Provided by Coriell	CD_50_ of Stx2 (ng/mL) (SEM)
GM17135	No disease reported	0.05
GM17139 ^b^	No disease reported	0.5
GM17154	No disease reported	0.08 (SEM 0.05)
GM17158	No disease reported	100 (SEM 50)
GM17197	No disease reported	0.06
GM17464 ^c^	No disease reported	>2000
GM17619	No disease reported	50 (SEM 23)
GM17645	No disease reported	130
GM17658	No disease reported	>500
GM00333	No disease reported	>500
GM00607	No disease reported	>500
GM04258	Severe combined immunodeficiency	2 (SEM 2.4)
GM03380	Ataxia-Telangiectasia	0.5 (SEM 0.16)
GM02473	Xeroderma pigmentosum	0.005
GM06989	CEPH/Utah pedigree 1328	0.07
GM07019	CEPH/Utah pedigree 1340	0.05
GM07014	CEPH/Utah pedigree 13292	50 (SEM 34)

^a^ Experiments were done with an n = 3–7, and the average is shown. ^b^ GM17139 n = 2 (no standard error [SEM] shown). ^c^ This cell line was also resistant to Stx1 and Stx2d.

## Data Availability

The original contributions presented in this study are included in the article/Supplementary Material. Further inquiries can be directed to the corresponding author.

## Supplementary Materials

The following supporting information can be downloaded at https://www.mdpi.com/article/10.3390/microorganisms14071505/s1, Figure S1: Susceptibility of B-lymphoblastoid cell lines to Stx1 and Stx2d. Table S1: MRM transitions for Gb3 lipid species.

## Abbreviations

The following abbreviations are used in this manuscript:

Stx	Shiga toxin
STEC	Stx-producing *Escherichia coli*
Gb3	Globotriaosylceramide
DoD	Department of Defense
IRB	Institutional Review Board
NOU	Notification of use
FBS	Fetal bovine serum
RPMI	Roswell Park Memorial Institute Media
CD_50_	Cytotoxic dose
PBS	Phosphate buffered saline
MTBE	Methyl tert-butyl ether extraction
DCM	Dichloromethane/methanol
MRM	Multiple reaction monitoring
DP	Declustering potential
EP	Entrance potential
CE	Collision energy
CXP	Collisional exit potential
CUR	Curtain gas
CAD	Collisional activation dissociation
IS	Ion spray voltage
TEM	Ion spray temperature
GS1	Nebulizing gas
GS2	Heating gas
MFI	Median fluorescent intensity
