# Supplementary material for: Susceptibility of Human B-Lymphoblastoid Cells to Shiga Toxin Intoxication Homologues
Source: Microorganisms. 2026 Jul 10;14(7):1505. doi: 10.3390/microorganisms14071505 (PMC13413790; doi:10.3390/microorganisms14071505)
Supplement: Supplementary file 1 [file microorganisms-14-01505-s001.zip › microorganisms-4369460-supplementary.pdf]

Supplemental Material

Supplemental Figure S1. Susceptibility of B-lymphoblastoid cell lines to Stx1 and Stx2d. The CD_{50} values are shown. The bar represents the geometric mean value. The error bars indicate the geometric standard deviation. Stx1 was not tested on GM03715. n=2-6. The CD_{50} values shown are in ng/100 µL, and the corresponding values in Table 1 were normalized to ng/mL.

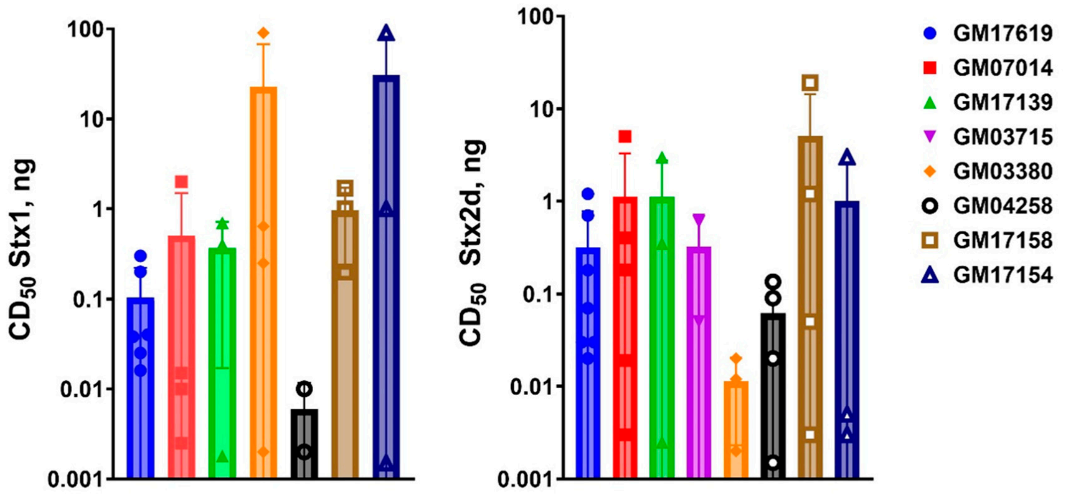

Supplemental Table S1. MRM transitions for Gb3 lipid species.

Q1	Q3	Gb3 Species
1026.7	266.6	Gb3 (18:0;O2/16:0)
1024.7	266.6	Gb3 (18:0;O2/16:1)
1040.7	266.6	Gb3 (18:0;O2/17:0)
1054.7	266.6	Gb3 (18:0;O2/18:0)
1052.7	266.6	Gb3 (18:0;O2/18:1)
1082.8	266.6	Gb3 (18:0;O2/20:0)
1080.7	266.6	Gb3 (18:0;O2/20:1)
1110.8	266.6	Gb3 (18:0;O2/22:0)
1108.8	266.6	Gb3 (18:0;O2/22:1)
1126.8	266.6	Gb3 (18:0;O2/22:0;O1)
1124.8	266.6	Gb3 (18:0;O2/22:1;O1)

1138.8	266.6	Gb3 (18:0;O2/24:0)
1136.8	266.6	Gb3 (18:0;O2/24:1)
1154.8	266.6	Gb3 (18:0;O2/24:0;O1)
1152.8	266.6	Gb3 (18:0;O2/24:1;O1)
1024.7	264.6	Gb3 (18:1;O2/16:0)
1022.7	264.6	Gb3 (18:1;O2/16:1)
1038.7	264.6	Gb3 (18:1;O2/17:0)
1052.7	264.6	Gb3 (18:1;O2/18:0)
1050.7	264.6	Gb3 (18:1;O2/18:1)
1080.7	264.6	Gb3 (18:1;O2/20:0)
1078.7	264.6	Gb3 (18:1;O2/20:1)
1108.8	264.6	Gb3 (18:1;O2/22:0)
1106.8	264.6	Gb3 (18:1;O2/22:1)
1124.8	264.6	Gb3 (18:1;O2/22:0;O1)
1122.8	264.6	Gb3 (18:1;O2/22:1;O1)
1136.8	264.6	Gb3 (18:1;O2/24:0)
1134.8	264.6	Gb3 (18:1;O2/24:1)
1152.8	264.6	Gb3 (18:1;O2/24:0;O1)
1150.8	264.6	Gb3 (18:1;O2/24:1;O1)
